# Pneumonic Plague in Johannesburg, South Africa, 1904

**DOI:** 10.3201/eid2401.161817

**Published:** 2018-01

**Authors:** Charles M. Evans, Joseph R. Egan, Ian Hall

**Affiliations:** University of Birmingham, Birmingham, UK (C.M. Evans);; Public Health England, Wiltshire, UK (J.R. Egan, I. Hall)

**Keywords:** bubonic plague, epidemic, epizootic, Ghandi, Johannesburg, pneumonic plague, rats, reproduction number, bacteria, zoonoses, South Africa

## Abstract

Plague is a potentially dangerous reemerging disease. Because modern outbreaks are relatively infrequent, data for epidemiologic study are best found in historical accounts. In 1905, the Rand Plague Committee published a report describing an explosive outbreak of 113 cases of pneumonic plague that occurred in Johannesburg, South Africa, in 1904. Using these data, we investigated social, spatial, and temporal dynamics and quantified transmissibility as measured by the time-varying reproduction number. Risk for transmission was highest when friends, family members, and caregivers approached the sick. Reproduction numbers were 2–4. Transmission rates rapidly diminished after implementation of control measures, including isolation and safer burial practices. A contemporaneous smaller bubonic plague outbreak associated with a low-key epizootic of rats also occurred. Clusters of cases of pneumonic plague were mostly limited to the Indian community; cases of bubonic plague were mostly associated with white communities and their black African servants.

Since the epidemics of primary pneumonic plague in Manchuria in the early 20th century, few opportunities for the study of substantial epidemics have occurred (*1*). Often small, self-limiting (*2*), and occurring in inaccessible places, epidemics may well have run their course before medical teams arrived (*3*). When authorities are vigilant, patients and contacts can be quickly identified, isolated, and, when possible, given effective antimicrobial drugs (*4*). Nevertheless, the pneumonic form of *Yersinia pestis* infection remains a potential threat to public health (*5*), and an aerosolized preparation might be used as a biological weapon (*6*). The death rate among patients who do not receive treatment approaches 100%, which makes pneumonic plague one of the most lethal diseases known to humanity, and lately, antimicrobial drug–resistant strains have been detected (*7*).

Historical records are therefore a useful source of data for epidemiologic studies. One such epidemic, which has received limited attention, occurred in 1904 in Johannesburg, South Africa, adjacent to the Witwatersrand gold fields. In 1905, the Rand Plague Committee published a report (the RPCR) that documented the principal findings together with the data on which their inferences were based (*8*). A copy of the RPCR can be found in the Wellcome Library, 183 Euston Road, London NW1 2BE, UK. It was compiled by Walter Pakes (acting for Charles Porter, Medical Officer of Health for Johannesburg) and comprises 103 pages of text accompanied by plates and folded maps. We reexamined these data in light of modern knowledge and recently developed analytical techniques.

## Materials and Methods

### Social, Political, and Spatial Context 

In 1886, the Witwatersrand gold fields opened, and Witwatersrand soon became the largest gold-producing region in the world. After the second Boer war, in May 1900 British forces occupied Johannesburg, achieving economic and political dominance. Up to and beyond 1904, the racial makeup of Johannesburg was determined by the need to attract highly skilled workers and cheap labor. From 1860 on, workers from India entered the Colony of Natal under a system of indenture organized by the British and white farmers, and from 1896, the Chamber of Mines coordinated the recruitment of black African labor through the Witwatersrand Native Labour Association (*9*).

**Figure 1:**
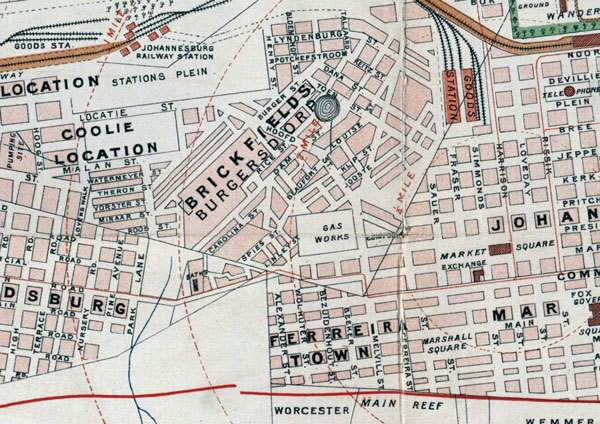
Central area of Johannesburg, South Africa, in 1904, showing the relative positions of the Coolie Location, Burgersdorp, and Market Square.  Map held at the Witwatersrand Library, University of the Witwatersrand, Johannesburg, South Africa, and available at http://innopac.wits.ac.za/search/?searchtype=t&SORT=D&searcharg=plan+of+johannesburg+and+suburbs

**Figure 2:**
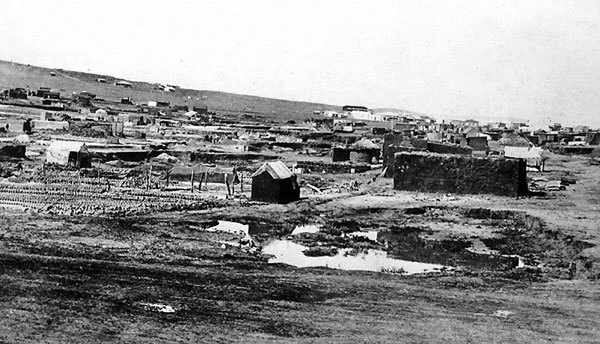
The Coolie Location. Photograph kindly supplied by Museum Africa, 121 Lilian Ngoyi (formerly Bree) St., Newtown, Johannesburg, South Africa.

After the completion of indenture, Indian workers were permitted to buy “preferent” rights to parcels of land in Johannesburg, known as “stands,” within a small area widely known as the Coolie Location (Figure 1). This area subsequently became a multiracial slum, causing the municipal authorities to repurchase it for redevelopment. Mahatma Gandhi made a name for himself by securing fair compensation for the Indian property owners (*10*). A photograph (Figure 2) depicts a ramshackle collection of dwellings, described as follows in the RPCR: “… although the Coolie Location is theoretically laid out in stands, the tin shanties were put in all sorts of positions… the whole of Stands 43 and 48 must be considered as one mass of little huts, with common latrines, etc where the inhabitants… were in very constant and close contact with… their neighbors.”

The central focus of Johannesburg was the market square, where the post office, stock exchange, banks, and shopping outlets were located. The market square received goods, which were often auctioned on site, from the coast and elsewhere. Within the square, buildings housed additional shops, restaurants, and offices. Between the Coolie Location and market square was an area known as Brickfields or Burgersdorp. Originally established by poor Afrikaner families, who fabricated sun-baked bricks to supply the gold-driven property boom, this area also become a multiracial slum (*11*).

African workers recruited or coerced into supplying cheap labor for the mines were housed in compounds close to their place of work but some distance from Johannesburg center (*12*). Conditions there were poor, and pneumonia was commonplace (*13*). The Johannesburg census of April 17, 1904, recorded 118,917 male and 39,663 female inhabitants (*14*). The racial distribution was described as follows: “Europeans/whites,” 52,042 male and 31,680 female; “all coloured races, including South African aboriginals and Asiatics,” 66,875 male and 7,803 female.

### The RPCR as Data Source

Outbreaks of primary pneumonic plague and bubonic plague together with an associated epizootic among rats were documented. Details were given of the procedures adopted by public health workers, methods used for the identification of *Y. pestis*, and postmortem examinations. Case types were defined as follows: “Pure pneumonic cases were those in which no buboes could be found, but in which there was definite broncho-pneumonia. The mixed cases were those in which there was definite broncho-pneumonia, as well as buboes, and the B. Pestis [sic] was recovered both from the foci in the lungs and from the bubo. The septicaemic cases were those without either signs of pneumonia or buboes.”

A list of cases recorded from March 20 on indicated the date of death, type of infection, race, sex, occupation, and residential location for each patient. The RPCR also contained a retrospective study of 37 cases (originally thought to be pneumonia but which had occurred before March 20). Also noted were the cases of a Dr. Marais and 9 other patients with pneumonic plague (former inhabitants of the Coolie Location, who had fled to areas outside Johannesburg). To illustrate putative transmission pathways, all cases of pneumonic plague were logged on a fold-out chart; we used those dates of death for our analysis.

### Estimation of the Time-Varying Reproduction Number for Cases of Primary Pneumonic Plague 

The transmissibility of primary pneumonic plague can be quantified by estimating the time-varying reproduction number, R_t_, defined as the average number of secondary infections resulting from an infectious person. We used a recently published method of estimating R_t_ (*15*), which requires a count of the observed number of patients who became symptomatic each day. However, the date of onset of many of the primary pneumonic plague cases could not be ascertained because the first indication was often the discovery of a deceased person and no witness to the date of disease onset. Thus, in our study, we back-calculated the incidence of symptom onset (*16*,*17*) from the death count by using the symptom onset to death distribution, based on 166 cases, previously estimated from the primary pneumonic plague outbreak in Manchuria (*1*). These data provide a mean (±SD) of 2.3 (±1.7) days. A second prerequisite for estimating R_t_ is the serial interval distribution (i.e., the time from symptom onset in a primary case-patient to symptom onset in a secondary case-patient). We used a distribution, based on 177 cases, previously estimated from 4 outbreaks that occurred during the 20th century (*18*), providing a mean of 5.4 (±3.0) days.

## Results

### Time Course of the Epidemic

The RPCR states, “On the evening of the 18th March, the District Surgeon, Dr. Mackenzie, was informed that a number of Indians were sick in the Coolie Location… Dr. Alexander visited the Location and found a number of Indians suffering apparently from Pneumonia… during the evening of the 18th March, Mr. Gandhi, Dr. Godfrey and Mr. Madenjit… removed all the sick Indians they could find to Stand 36, Coolie Location…” Other reports suggest that Gandhi and his colleagues were the first to raise the alarm. William Godfrey, a doctor of Indian descent who graduated in 1903 from Edinburgh University (*19*), realized that plague had broken out and arranged for an empty house to be made available before a larger temporary hospital was later established. After bacteriologic confirmation of the presence of *Y. pestis*, a cordon was placed around the Coolie Location on March 20.

**Figure 3:**
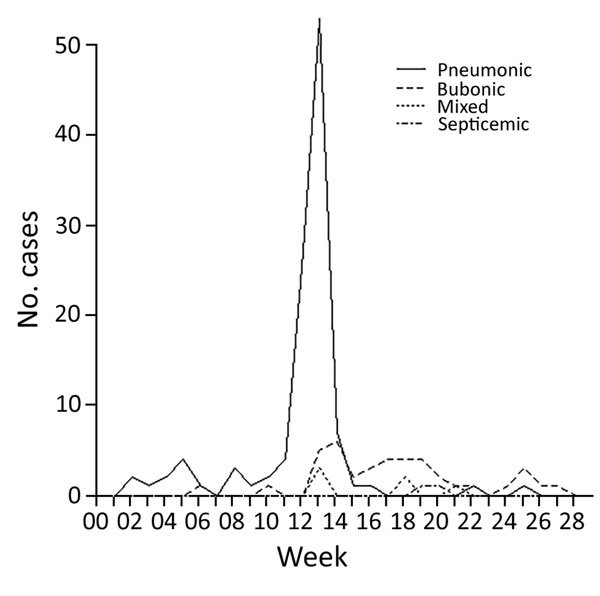
Incidence of the 4 types of plague over the duration of the epidemic in Johannesburg, South Africa, from week ending January 2 to week ending June 16, 1904.

The first cases of pneumonic plague to be documented in the retrospective study occurred between early January and mid-March (Figure 3). Two cases of bubonic plague occurred in the weeks ending February 6 and March 5 and were mentioned as follows: “A very careful search through the death returns for several months before the 18th March, 1904, fails to reveal any probable case of bubonic plague (as distinguished from pneumonic plague) with the exception of Cases VII and XIV...” Both patients were attended by medical officers. Inguinal swellings were noted, although a diagnosis of bubonic plague was not made at that time. Death occurred within 2 days of the examinations, but the report maintains that no further cases seem to have arisen from them.

The pneumonic phase of the epidemic showed an “explosive” increase during the 12th and 13th weeks, whereas the bubonic cases peaked in week 14 and continued over the next 14 weeks. We note that pneumonic plague cases were recorded on a date-of-death basis, whereas bubonic plague cases were recorded on an onset-of-symptom basis. The investigators were unable to trace the index case(s) and could only speculate that “Plague infected rice was imported from Bombay during December, 1903 and January 1904. From this rice a few Indians were infected with the pneumonic form of plague.”

### Case Incidence

The 113 cases of pneumonic plague far outnumbered the 40 bubonic, 6 mixed, and 2 septicemic plague cases (Table). The racial group most severely hit by primary pneumonic plague was Indians, even though they were a small minority within Johannesburg. Of the 40 bubonic cases recorded, 16 were in “whites,” 14 in “natives,” 4 in “coloureds,” but only 6 were in “Indians.” Relatively few cases in girls/women were reported, and parity between the sexes was approached by the white group only. Survival was reported for 31 patients with bubonic plague but only 2 with pneumonic plague.

**Table:** Plague cases by disease type and patient race and sex, Johannesburg, South Africa, 1904*

Disease type	White			Indian			Colored			Native		Total
	M	F		M	F		M	F		M	F	
Pneumonic												
Cases	4	4		88	4		0	0		12	1	113
Deaths	4	4		86	4		0	0		12	1	111
Bubonic												
Cases	10	6		6	0		3	1		14	0	40
Deaths	1	2		3	0		0	0		3	0	9
Mixed												
Cases	0	0		3	0		0	0		3	0	6
Deaths	0	0		3	0		0	0		3	0	6
Septicemic												
Cases	2	0		0	0		0	0		0	0	2
Deaths	2	0		0	0		0	0		0	0	2
Total												
Cases	16	10		97	4		3	1		29	1	161
Deaths	7	6		92	4		0	0		18	1	128

*The actual number of deaths from pneumonic plague recorded was 111 and not 67 as reported in Table 2, page 12, of the Rand Plague Committee report (*8*). Similarly, the number of deaths from bubonic infection was 9 and not 7. The text of the RPCR indicates that the category “coloured” refers to “coloured people other than Indians and Natives,” but further clarification is not provided.

### Public Health Measures

In addition to the isolation of patients and the establishment of a cordon, inspectors were appointed to search the Coolie Location for additional sick persons. Within the Witwatersrand, every compound housing native mineworkers was eventually inspected and the movement of natives was controlled by the existing native pass system. Pneumonia was made a notifiable disease, and samples of sputum were routinely sent to government laboratories.

On April 8, the authorities razed the Coolie Location by burning it to the ground. The inhabitants, including “1,600 Asiatics, 142 coloured and 1,358 natives,” were relocated to a camp 12 miles from the center of Johannesburg, the first step in the evolution of the township later to become known as Soweto (*20*).

We found no record of any special protective clothing or instructions being issued to those caring for primary pneumonic plague patients. A single nurse was allocated to the temporary hospital, but the Indian orderlies were left to their own devices. The RPCR also mentions that a nurse allocated to look after infected white children “…treated the pneumonic cases as bubonic cases. Certainly she kissed the children.”

As the outbreak progressed, most deaths occurred in hospitals that allowed some control of burial practices. The RCPR states that “… in the case of Hindoos and the Mohammedans [sic]. The former were allowed to bury their dead: the latter, who have certain religious functions to perform were given a room in the mortuary to perform the rite. They were warned of the dangers of handling the cadavers, and it was suggested to them that the washing should be performed with a solution of corrosive sublimate.”

### Examinations for Bacteria and Pathologic Findings 

Government health laboratories tested for *Y. pestis* in samples of sputum or tissues from organs including the lung, spleen, and liver. Bacteria were cultured, identified morphologically, and subsequently confirmed by inoculation into rabbits and guinea pigs.

The RPCR claims that postmortem examinations were conducted on every patient whose death was suspicious throughout the Rand. Before death, primary pneumonic plague patients exhibited typical symptoms of fever and some “scanty but blood-stained expectorations.” Death occurred generally within 72 hours of symptom onset, and postmortem appearances were described as follows: “… signs of pleurisy were common and… extensive fibrinous exudation... B. Pestis [sic] was very abundantly present… but… not… sufficient pneumonic lung to account for death… in some… cases death is due to toxaemia and not to septicaemia.”

### Time-Varying R_t_ for Cases of Primary Pneumonic Plague and Effectiveness of Public Health Measures

**Figure 4:**
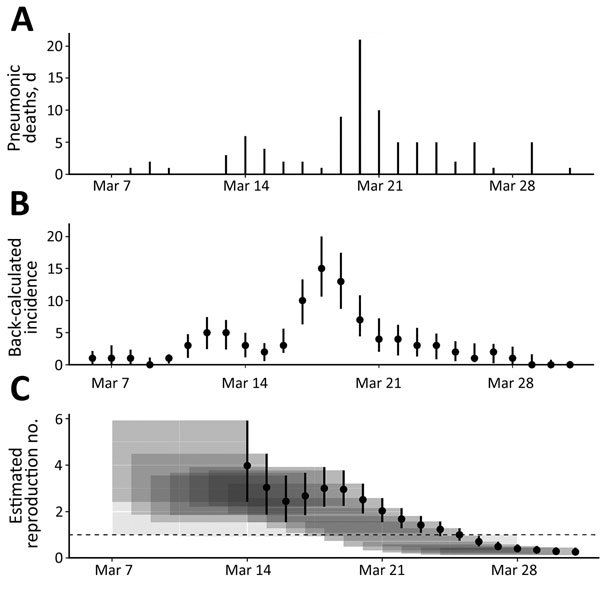
A) Deaths per day resulting from primary pneumonic plague in Johannesburg, South Africa, March 7–31, 1904. B) Back-calculated number of case-patients experiencing symptom onset. Circles represent most likely values; error bars represent 95% CIs. C) Transmissibility of primary pneumonic plague as measured by reproduction number, R_t_. Circles represent the most likely values, error bars represent 95% CIs, and shaded polygons represent the period over which R_t_ was estimated. Uncertainty in the back-calculated incidence has not been accounted for in the transmission estimates, which means that the variations in the time-varying R_t_ are probably underestimated because the incidence curve is smoothed out somewhat by the back-calculation process (and also reduced slightly because of rounding to the nearest integer). However, because the 7-day sliding window has the effect of smoothing out the R_t_ estimates in any case, not accounting for the uncertainty in the back-calculation probably has a limited effect on panel C results.

The number of deaths from pneumonic plague and back-calculated incidence for the explosive period in March are shown in Figure 4, panels A and B, respectively; Figure 4, panel C, shows the corresponding estimates of the time-varying Rt using a 7-day sliding window over which the transmission is estimated. The first estimate of R_t_ provided on March 14 covers all estimated dates of symptom onset from March 7 (i.e., the preceding week). The small numbers of estimated dates of onset before this time prevented our estimating transmission with any confidence; indeed, confidence intervals are much larger in the early stages of the outbreak. The fall and then rise in transmissibility that occurred March 14–19 reflect a similar pattern in the estimated incidence, giving R_t_ estimates of ≈2–4. As a consequence of the decreasing estimated incidence over the later part of March, the transmission estimates also decreased. By March 26, R_t_ decreased to <1, indicating that transmission levels were not sustainable and the outbreak was ending. The decrease in estimated transmissibility coincides with the start of the isolation process on March 18, suggesting that this strategy was probably effective.

Other factors that may also have contributed to the decrease in R_t_ include the local depletion of susceptible persons, because the primary pneumonic plague outbreak was manifestly confined to a limited number of dwellings within the Coolie Location and Brickfields, while others, prevented from fleeing, may well have sought to avoid contact with sick persons. The effectiveness of the cordon surrounding the Coolie Location is questionable because we have on record that 9 persons fled the area and died elsewhere. This action, and the later razing of the Coolie Location, is best thought of as part of a plan to allay the fears of white citizens.

### Clustering of Primary Pneumonic Plague Cases

Of the 8 white persons with primary pneumonic plague, 7 formed a distinct cluster, including Dr. Marais, his wife, and 3 of his children living in the Fordsburg district. While nursing the Marais family, a female nurse and a male clerk acquired the disease. Within the Coolie Location, 45 of 64 cases were recorded in only 8 of 96 stands. Stand 93 housed 16 case-patients, stand 47 housed 7 case-patients, and stand 48 housed 5 case-patients. Similarly, of 20 cases recorded in the extensive Brickfields/Burgersdorp area, 15 were located at only 3 addresses. Investigations of the Indian community identified 16 probable transmissions involving nursing, preparing bodies for funerals, attending funerals, or close family members. There was a considerable amount of social contact between residents of the Brickfields stands and stand 93 in the Coolie Location. The RPCR (p. 47) notes that “Among the inhabitants of Stands 47 and 93 were many fruit hawkers, and some of these were not only of the same caste, but actually came from the same village in India as the Indians employed at the S.A. Fruit Store… [inhabitants of Stand 618, Burgersdorp]… There is little doubt that they met one another at Stand 93.”

In both communities, transmission seemed to follow relationship pathways involving intimate contact. However, there is no evidence that those who fled (some as far as Durban) transmitted the disease to others.

### The Epizootic

The RPCR (p. 58) states that a municipal rat catcher was appointed on August 5, 1903, and from that date until March 17, 1904, a total of 8,972 rats were caught. During December 27, 1903–March 18, 1904, a total of 160 rats were sent to government laboratories, but only 13 (11.4%) of 114 examined were positive for *Y. pestis*. During March 18–July 31, 1904, a total of 1,657 rats were sent but only 95 (6.0%) of 1,583 examined were positive. Rats that were not examined had been mummified or were in an advanced state of putrefaction. There is no record of any investigation concerning rat fleas, and the species of rat was not identified, suggesting that the investigators did not yet appreciate the role of fleas in plague transmission.

The market buildings, post office, native pass office, and at least 4 restaurants and hotels near the market square were found to be infested with rats. The RPCR comments, “Upon pulling down the inside wooden lining, a terrible condition was found. Rat runs were seen throughout the [Market] buildings and a large number of dead and mummified rats were found…”

However, investigators consistently found little evidence of rats in the Coolie Location. For example, the RPCR states, “… during the course of the year 1903… the inhabitants of the Coolie Location stated most emphatically that there had been no rats in the location for some considerable time before the outbreak and the comparative absence of rats was abundantly proved… a well organized search was made, and as the result, not a dozen rats in all were found or caught.”

Although rats were much more likely to be found near the market square than within the Coolie Location, a complete absence of rats in the location was unlikely. The RPCR states, “Tom Nesabi, a Basuto, was in the employ of a store-keeper at Stand 12… Tom did all the rough work in the store, carrying stacks of rice, cleaning up the shop and so on. His employer states that there were no rats in the store, but his mother states that Tom had a dog which used to kill the rats and the dog died shortly before Tom.”

### The Connection between the Epizootic and the Bubonic Plague Outbreak

**Figure 5:**
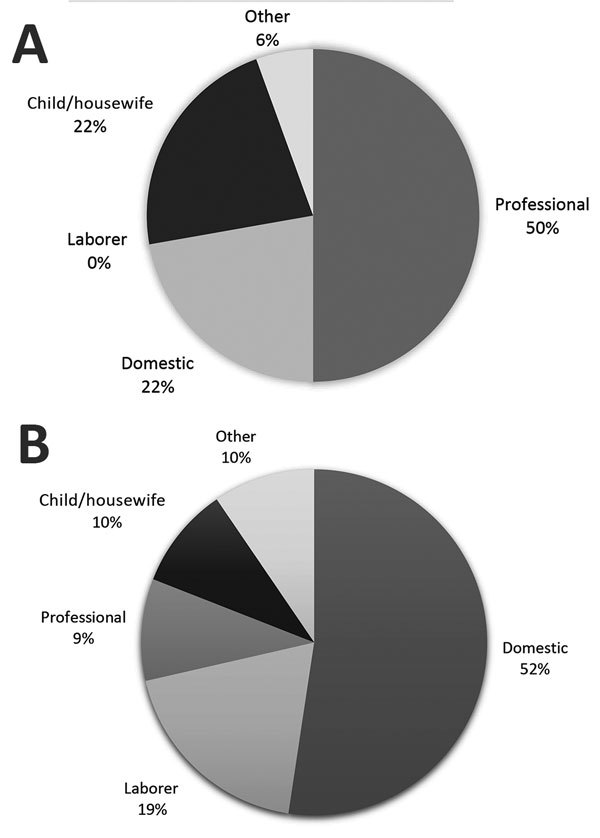
Occupations of white (A) and native (B) patients with bubonic, mixed, or septicemic plague, Johannesburg, South Africa, 1904.

In Johannesburg in 1904, a total of 48 cases of bubonic, mixed, and septicemic plague were reported. No information was available for 4 case-patients, but of the remainder, 15 (34%) lived or worked in buildings where rats were found and another 14 (32%) lived or worked in buildings where rats infected by *Y. pestis* were found. Furthermore, 26 (90%) case-patients associated with rats lived or worked within one half mile of the market buildings. Figure 5 illustrates the occupations of the white and native case-patients with bubonic, mixed, or septicemic plague. Most white case-patients were professional men and their family members, whereas more than half of the native case-patients worked as domestic employees.

## Discussion

Arguably, the performance of the Johannesburg authorities in 1904 should be judged by the standards of the day, when antimicrobial agents were not available and the role of fleas as vectors had not yet been firmly established. Nevertheless, the standard of evidence presented in the RPCR was sufficient for us now to demonstrate that social, spatial, and environmental factors helped shape this epidemic.

A particularly noteworthy aspect of this outbreak of primary pneumonic plague was that none of the 9 escapees from the Coolie Location transmitted the disease to the wider population; the RPCR also lists lack of transmission by many other case-patients. Nevertheless, within social networks characterized by family connections, employment, caste, and so on, the disease spread rapidly. Recent outbreaks recorded in India (*21*) and China (*4*) followed this pattern in which the disease is transmitted to relatives, friends, or caregivers but not to more loosely associated contacts. The relative paucity of female patients is best explained by the preponderance of men attracted by the gold rush opportunity.

The fact that the explosive phase of the outbreak was surprisingly short-lived also deserves special attention. It seems improbable that *Y. pestis* could lose its virulence over 2 weeks, and we should look to the behavior of potential contacts and the implementation of public health measures. Here, the value of isolating infected persons was immediately appreciated by Dr. Godfrey and Mahatma Gandhi, and progress was made before municipal authorities first realized that the epidemic was fully under way.

The investigators were unable to identify the index case-patient(s) who initiated the outbreak of pneumonic plague, and there seems little chance of retrospectively doing so from the content of the RPCR. There is little evidence to confirm the conventional view that such cases originated through airborne transmission from patients with bubonic plague in whom secondary pneumonic plague had developed (mixed cases) and no evidence that a person from outside Johannesburg introduced pneumonic plague into the area.

The later outbreak of bubonic plague was the likely consequence of a modest epizootic that started in the previous year. Cases of bubonic plague were found in the environs of the market square and mainly involved white professionals and their families along with their black African servants. Surprisingly, the percentage of case-patients with pure bubonic plague who recovered was relatively high, which might suggest that this strain of *Y. pestis* was not especially virulent. However, against this argument, we have evidence that, when transmitted via the pneumonic route, the disease was almost 100% fatal.

It has been argued elsewhere that primary pneumonic plague is not as transmissible as some suppose (*22*). Indeed, it is well known that primary pneumonic plague rapidly incapacitates the patient, who is then incapable of reaching potential contacts within the most infectious period. Nevertheless, this study shows that relatively high rates of transmission were achieved in Johannesburg in 1904, as demonstrated by the peak values for the estimated time-varying R_t_. These high rates are probably the consequence of crowding and spread through social networks, which facilitated transmission in a similar way to that observed in West Africa during the recent epidemic of Ebola virus disease (*23*), which is also not easily transmitted. In conclusion, we suggest that the pneumonic form of plague also remains a potentially serious threat in locations that are relatively inaccessible or that have limited capacity for a robust public health response. 
